# Supplementary material for: The degree of internationalization of Chinese Multinationals along the belt and road initiative countries
Source: PLoS One. 2020 Jul 30;15(7):e0236636. doi: 10.1371/journal.pone.0236636 (PMC7392271; doi:10.1371/journal.pone.0236636)
Supplement: S1 Appendix — (DOCX) [file pone.0236636.s001.docx]

**S1 Appendix. List of Firms with their respective scores for all variables.**

| COMPANY | TYPE | DOI | FSTS | OSTS | PDIO | GD | AGE |
| --- | --- | --- | --- | --- | --- | --- | --- |
| Qingjian | POE | 2.111 | 0.999 | 1.000 | 0.100 | 0.012 | 9 |
| Texhong Textile | POE | 2.081 | 0.969 | 1.000 | 0.100 | 0.012 | 19 |
| Qingdao Hengshun Zhongsheng | SOE | 1.809 | 0.975 | 0.722 | 0.100 | 0.012 | 9 |
| Geo-Jade Petroleum | POE | 1.433 | 0.898 | 0.423 | 0.100 | 0.012 | 22 |
| Zhongman Petroleum | POE | 1.383 | 0.882 | 0.389 | 0.100 | 0.012 | 34 |
| Huawei | POE | 1.382 | 0.501 | 0.545 | 0.300 | 0.036 | 33 |
| Sailun Group | POE | 1.348 | 0.710 | 0.526 | 0.100 | 0.012 | 16 |
| Tempus | POE | 1.302 | 0.591 | 0.600 | 0.100 | 0.012 | 22 |
| China Aerospace Science and Technology | SOE | 1.246 | 0.522 | 0.500 | 0.200 | 0.024 | 19 |
| Sanghai Safbon | POE | 1.243 | 0.710 | 0.421 | 0.100 | 0.012 | 12 |
| Sino Great Wall | POE | 1.234 | 0.495 | 0.344 | 0.300 | 0.095 | 17 |
| Beijing Enterprises | SOE | 1.224 | 0.000 | 1.000 | 0.200 | 0.024 | 24 |
| China Singyes | POE | 1.193 | 0.081 | 1.000 | 0.100 | 0.012 | 23 |
| Cybernaut | POE | 1.176 | 0.564 | 0.500 | 0.100 | 0.012 | 9 |
| Power Construction Corp | SOE | 1.175 | 0.236 | 0.034 | 0.500 | 0.405 | 8 |
| China Nonferrous | SOE | 1.172 | 0.393 | 0.667 | 0.100 | 0.012 | 9 |
| China Communications Construction | SOE | 1.145 | 0.212 | 0.088 | 0.500 | 0.345 | 19 |
| China Fortune | POE | 1.112 | 0.000 | 1.000 | 0.100 | 0.012 | 22 |
| Country Garden Holdings | POE | 1.112 | 0.000 | 1.000 | 0.100 | 0.012 | 24 |
| Bank of China | SOE | 0.992 | 0.229 | 0.538 | 0.200 | 0.024 | 30 |
| China Mobile | SOE | 0.981 | 0.000 | 0.833 | 0.100 | 0.048 | 24 |
| China International Marine Containers | SOE | 0.962 | 0.564 | 0.286 | 0.100 | 0.012 | 19 |
| Geely Auto | POE | 0.934 | 0.022 | 0.800 | 0.100 | 0.012 | 8 |
| ICBC | SOE | 0.927 | 0.123 | 0.692 | 0.100 | 0.012 | 17 |
| CNPC | SOE | 0.917 | 0.332 | 0.167 | 0.300 | 0.119 | 14 |
| Xinyi Glass | POE | 0.887 | 0.275 | 0.500 | 0.100 | 0.012 | 35 |
| Guangdong Midea | POE | 0.885 | 0.402 | 0.259 | 0.200 | 0.024 | 33 |
| Alibaba | POE | 0.824 | 0.000 | 0.652 | 0.100 | 0.071 | 19 |
| Lifan | POE | 0.811 | 0.437 | 0.262 | 0.100 | 0.012 | 22 |
| Shandong Nanshan Aluminum | POE | 0.796 | 0.284 | 0.400 | 0.100 | 0.012 | 20 |
| China Energy Engineering | SOE | 0.768 | 0.154 | 0.000 | 0.400 | 0.214 | 13 |
| China Railway | SOE | 0.768 | 0.051 | 0.043 | 0.400 | 0.274 | 18 |
| Sinopec | SOE | 0.767 | 0.226 | 0.133 | 0.300 | 0.107 | 10 |
| Zhejiang Jinke | POE | 0.757 | 0.478 | 0.167 | 0.100 | 0.012 | 23 |
| Guizhou Tyre | SOE | 0.756 | 0.445 | 0.200 | 0.100 | 0.012 | 18 |
| Jinko Solar | POE | 0.731 | 0.619 | 0.000 | 0.100 | 0.012 | 17 |
| ZTE | SOE | 0.727 | 0.440 | 0.163 | 0.100 | 0.024 | 17 |
| State Construction Engineering | SOE | 0.716 | 0.078 | 0.000 | 0.400 | 0.238 | 32 |
| Dongfang Electric | SOE | 0.706 | 0.096 | 0.150 | 0.400 | 0.060 | 7 |
| China National Offshore Oil | SOE | 0.697 | 0.300 | 0.286 | 0.100 | 0.012 | 16 |
| Sany Heavy | POE | 0.693 | 0.364 | 0.217 | 0.100 | 0.012 | 23 |
| Harbin Electric | SOE | 0.669 | 0.309 | 0.000 | 0.300 | 0.060 | 12 |
| Shanghai Electric | SOE | 0.644 | 0.112 | 0.061 | 0.400 | 0.071 | 20 |
| Tebian Electric Apparatus | POE | 0.632 | 0.180 | 0.069 | 0.300 | 0.083 | 24 |
| China Railway Construction | SOE | 0.618 | 0.052 | 0.000 | 0.400 | 0.167 | 29 |
| China Galaxy Securities | POE | 0.591 | 0.018 | 0.462 | 0.100 | 0.012 | 12 |
| ZOJE Resources | POE | 0.583 | 0.380 | 0.091 | 0.100 | 0.012 | 27 |
| Fosun | POE | 0.578 | 0.466 | 0.000 | 0.100 | 0.012 | 16 |
| Hisense Group | SOE | 0.577 | 0.315 | 0.150 | 0.100 | 0.012 | 7 |
| China National Chemical Engineering | SOE | 0.577 | 0.175 | 0.130 | 0.200 | 0.071 | 9 |
| Jiangsu Sunshine | POE | 0.544 | 0.341 | 0.091 | 0.100 | 0.012 | 19 |
| China National Building Material | SOE | 0.542 | 0.022 | 0.125 | 0.300 | 0.095 | 17 |
| Yuanda | POE | 0.527 | 0.253 | 0.162 | 0.100 | 0.012 | 18 |
| Qingdao Doublestar | SOE | 0.497 | 0.340 | 0.045 | 0.100 | 0.012 | 6 |
| Xuzhou Construction Machinery | SOE | 0.464 | 0.147 | 0.205 | 0.100 | 0.012 | 7 |
| Tencent | POE | 0.462 | 0.045 | 0.158 | 0.200 | 0.060 | 10 |
| Beijing Capital | SOE | 0.419 | 0.290 | 0.018 | 0.100 | 0.012 | 8 |
| LONGi Green Energy | POE | 0.409 | 0.245 | 0.040 | 0.100 | 0.024 | 20 |
| Shandong Ruyi | POE | 0.403 | 0.291 | 0.000 | 0.100 | 0.012 | 19 |
| China Western Power Industrial | POE | 0.397 | 0.142 | 0.143 | 0.100 | 0.012 | 23 |
| HNA | SOE | 0.388 | 0.164 | 0.000 | 0.200 | 0.024 | 16 |
| Zijin Mining | SOE | 0.386 | 0.062 | 0.200 | 0.100 | 0.024 | 17 |
| Power Construction Corp, HTG | SOE | 0.382 | 0.236 | 0.034 | 0.100 | 0.012 | 17 |
| Anhui Conch | SOE | 0.348 | 0.010 | 0.114 | 0.200 | 0.024 | 19 |
| Great Wall Motor | POE | 0.343 | 0.018 | 0.213 | 0.100 | 0.012 | 17 |
| BAIC | SOE | 0.335 | 0.000 | 0.111 | 0.200 | 0.024 | 23 |
| Xinjiang Beiken Energy Engineering | POE | 0.334 | 0.000 | 0.222 | 0.100 | 0.012 | 55 |
| MCC | SOE | 0.308 | 0.065 | 0.083 | 0.100 | 0.060 | 17 |
| SAIC | SOE | 0.271 | 0.034 | 0.114 | 0.100 | 0.024 | 5 |
| Huaxin Cement | POE | 0.271 | 0.077 | 0.082 | 0.100 | 0.012 | 11 |
| Ctrip | POE | 0.230 | 0.000 | 0.118 | 0.100 | 0.012 | 12 |
| Shandong Gaosu | SOE | 0.224 | 0.000 | 0.000 | 0.200 | 0.024 | 9 |
| China Ocean Shipping | SOE | 0.223 | 0.000 | 0.111 | 0.100 | 0.012 | 22 |
| Zhuzhou Kibing | POE | 0.219 | 0.024 | 0.083 | 0.100 | 0.012 | 23 |
| Shenhua | SOE | 0.214 | 0.015 | 0.075 | 0.100 | 0.024 | 16 |
| Hebei Iron | SOE | 0.210 | 0.022 | 0.077 | 0.100 | 0.012 | 28 |
| Gosun holding | POE | 0.195 | 0.006 | 0.077 | 0.100 | 0.012 | 22 |
| Zhuhai Port Holdings | SOE | 0.194 | 0.058 | 0.024 | 0.100 | 0.012 | 23 |
| Guangzhou R&F Properties | POE | 0.185 | 0.009 | 0.064 | 0.100 | 0.012 | 22 |
| Shanghai Tunnel Engineering | SOE | 0.185 | 0.053 | 0.019 | 0.100 | 0.012 | 23 |
| China Tianying* | POE | 0.172 | 0.000 | 0.060 | 0.100 | 0.012 | 18 |
| Shandong Landbridge | SOE | 0.160 | 0.048 | 0.000 | 0.100 | 0.012 | 24 |
| China Animal Husbandry | SOE | 0.158 | 0.046 | 0.000 | 0.100 | 0.012 | 17 |
| Shanghai Greenland | POE | 0.154 | 0.010 | 0.020 | 0.100 | 0.024 | 9 |
| Xinjiang Communications Construction | SOE | 0.152 | 0.001 | 0.040 | 0.100 | 0.012 | 6 |
| Anbang | POE | 0.134 | 0.011 | 0.000 | 0.100 | 0.024 | 20 |
| Jumei | POE | 0.112 | 0.000 | 0.000 | 0.100 | 0.012 | 6 |
| Shanghai international Airport | SOE | 0.112 | 0.000 | 0.000 | 0.100 | 0.012 | 18 |
| Shanghai Shentong | SOE | 0.112 | 0.000 | 0.000 | 0.100 | 0.012 | 24 |
| Yunnan Energy Investment | SOE | 0.112 | 0.000 | 0.000 | 0.100 | 0.012 | 14 |
| Zhongrun Resources | POE | 0.112 | 0.000 | 0.000 | 0.100 | 0.012 | 28 |
