# Supplementary material for: The degree of internationalization of Chinese Multinationals along the belt and road initiative countries
Source: PLoS One. 2020 Jul 30;15(7):e0236636. doi: 10.1371/journal.pone.0236636 (PMC7392271; doi:10.1371/journal.pone.0236636)
Supplement: S2 Appendix — (DOCX) [file pone.0236636.s002.docx]

**S2 Appendix. List of BRI countries where firms have invested and the corresponding number of Psychic Zones**

| **Company** | **Type** | **# BRI Countries Where Present** | **# Ronen And Shenkar Psychic Zones** |
| --- | --- | --- | --- |
| Alibaba | POE | India, Pakistan, Singapore, Thailand, South Korea, India, Singapore | 1 |
| Anbang | POE | South Korea | 1 |
| Anhui Conch | SOE | Cambodia Russia Indonesia | 2 |
| BAIC | SOE | South Africa , India | 2 |
| Bank of China | SOE | Turkey , Singapore | 2 |
| Beijing Capital | SOE | New Zealand | 1 |
| Beijing Enterprises | SOE | Russia, Malaysia | 2 |
| China Aerospace Science and Technology | SOE | Bolivia , South Korea | 2 |
| China Animal Husbandry | SOE | New Zealand | 1 |
| China Communications Construction | SOE | Afghanistan, Bangladesh, Bolivia, Brunei, Cambodia, Croatia, Ethiopia, Indonesia, Iran, Iraq , Israel, Kyrgyzstan, Latvia, Madagascar, Malaysia, Maldives, Montenegro, Myanmar, Pakistan, Panama, Papua New Guinea, Philippines, Qatar , Saudi Arabia, Serbia, Singapore, Sri Lanka, Thailand, Trinidad and Tobago, Yemen | 5 |
| China Energy Engineering | SOE | Bangladesh , Belarus, Bosnia, Egypt, Iran , Jordan, Kazakhstan, Kuwait, Malaysia, Mongolia, Myanmar, Pakistan, Philippines, Qatar, Russia, Ukraine, Vietnam. | 4 |
| China Fortune | POE | Indonesia | 1 |
| China Galaxy Securities | POE | Malaysia | 1 |
| China International Marine Containers | SOE | Singapore | 1 |
| China Mobile | SOE | Pakistan, South Korea, Thailand | 1 |
| China National Building Material | SOE | Croatia, Egypt ,India, Indonesia, Pakistan, Saudi-Arabia, Ukraine, Uzbekistan | 3 |
| China National Chemical Engineering | SOE | Georgia, Indonesia, Kazakhstan , Malaysia, Pakistan, Russia, | 2 |
| China National Offshore Oil | SOE | Qatar | 1 |
| China Nonferrous | SOE | Kazakhstan | 1 |
| China Ocean Shipping | SOE | UAE | 1 |
| China Railway | SOE | Bangladesh, Cambodia, Egypt, Ethiopia, Hungary, Israel, Kazakhstan , Kuwait, Malaysia, Maldives, Mongolia, Morocco, Nepal, Pakistan, Papua New Guinea, Philippines, Russia, Serbia, Singapore, Timor Leste, Uzbekistan, Vietnam | 4 |
| China Railway Construction | SOE | Malaysia, Bolivia, Saudi Arabia, Antigua and Barbuda, Egypt, Pakistan, Indonesia ,Ethiopia, Qatar, Thailand, Russia, Bangladesh, Malaysia, Iran, Pakistan, Turkey | 4 |
| China Singyes | POE | Uzbekistan | 1 |
| China Tianying* | POE | Vietnam | 1 |
| China Western Power Industrial | POE | Thailand | 1 |
| CNPC | SOE | Bangladesh,Iran,Iraq,Kyrgyzstan,Malaysia,Russia,Saudi,Turkmenistan, UAE, Uzbekistan | 3 |
| Country Garden Holdings | POE | Malaysia | 1 |
| Ctrip | POE | India | 1 |
| Cybernaut | POE | Russia | 1 |
| Dongfang Electric | SOE | Bosnia, Turkey, Egypt, Ethiopia, Georgia | 4 |
| Fosun | POE | India | 1 |
| Geely Auto | POE | Malaysia | 1 |
| Geo-Jade Petroleum | POE | Kazakhstan | 1 |
| Gosun holding | POE | SIngapore | 1 |
| Great Wall Motor | POE | Russia | 1 |
| Guangdong Midea | POE | India, Israel | 2 |
| Guangzhou R&F Properties | POE | Malaysia | 1 |
| Guizhou Tyre | SOE | Vietnam | 1 |
| Harbin Electric | SOE | Indonesia, Pakistan, Russia, UAE, Vietnam | 3 |
| Hebei Iron | SOE | Serbia | 1 |
| Hisense Group | SOE | Slovenia | 1 |
| HNA | SOE | Austria, Singapore | 2 |
| Huawei | POE | India, Serbia, New Zealand, Israel | 3 |
| Huaxin Cement | POE | Nepal | 1 |
| ICBC | SOE | Turkey | 1 |
| Jiangsu Sunshine | POE | Ethiopia | 1 |
| Jinko Solar | POE | Malaysia | 1 |
| Jumei | POE | South Korea | 1 |
| Lifan | POE | Russian | 1 |
| LONGi Green Energy | POE | India | 1 |
| MCC | SOE | Indonesia, Iran, India, Russia, Singapore | 1 |
| Power Construction Corp | SOE | Bangladesh,Bolivia,Bosnia,Egypt,Georgia,India,Indonesia,Iran,Israel,Jordan,Kazakhstan,Kuwait,Laos,Macedonia,Madagascar,Malaysia,Mongolia,Myanmar,Nepal,Oman,Pakistan,Philippines,Poland,Qatar,Russia,Saudi Arabia, Serbia Singapore, Sri Lanka, Thailand, Trinidad-Tobago, Vietnam | 5 |
| Power Construction Corp, HTG | SOE | Bangladesh | 1 |
| Qingdao Doublestar | SOE | South Korea | 1 |
| Qingdao Hengshun Zhongsheng | SOE | Philippines | 1 |
| Qingjian | POE | Singapore | 1 |
| SAIC | SOE | Malaysia, Indonesia | 1 |
| Sailun Group | POE | Vietnam | 1 |
| Sanghai Safbon | POE | Kazakhstan | 1 |
| Sany Heavy | POE | India | 1 |
| Shandong Gaosu | SOE | Bosnia, Timor leste | 2 |
| Shandong Landbridge | SOE | Panama | 1 |
| Shandong Nanshan Aluminum | POE | Indonesia | 1 |
| Shandong Ruyi | POE | Pakistan | 1 |
| Shanghai Electric | SOE | Egypt, Ethiopia, Malaysia, Saudi-Arabia, Serbia, UAE | 4 |
| Shanghai Greenland | POE | Malaysia, South Korea | 1 |
| Shanghai international Airport | SOE | Israel | 1 |
| Shanghai Shentong | SOE | Singapore | 1 |
| Shanghai Tunnel Engineering | SOE | Singapore | 1 |
| Shenhua | SOE | Indonesia, Russia, | 1 |
| Sino Great Wall | POE | Cambodia, Indonesia, Kuwait, Laos, Malaysia, Myanmar, Qatar, Sri Lanka | 3 |
| Sinopec | SOE | Ethiopia, Iran, Kazakhstan, Kuwait, Malaysia, Russia, Saudi Arabia, South Africa, Thailand | 3 |
| State Construction Engineering | SOE | Bangladesh, Brunei, Cambodia, Egypt, Ethiopia, Indonesia, Kuwait, Malaysia, Pakistan, Panama, Papua New Guinea, Russia, Singapore, Sri Lanka, UAE, Cambodia, South Korea, Maldives, Thailand, Vietnam, | 4 |
| Tebian Electric Apparatus | POE | Kyrgyzstan, India, Pakistan, Bangladesh, Ethiopia, Mongolia, Egypt | 3 |
| Tempus | POE | Maldives | 1 |
| Tencent | POE | India, Indonesia, Israel, South Korea, | 2 |
| Texhong Textile | POE | Vietnam | 1 |
| Xinjiang Beiken Energy Engineering | POE | Ukraine | 1 |
| Xinjiang Communications Construction | SOE | Ukraine | 1 |
| Xinyi Glass | POE | Malaysia | 1 |
| Xuzhou Construction Machinery | SOE | India | 1 |
| Yuanda | POE | Singapore | 1 |
| Yunnan Energy Investment | SOE | Laos | 1 |
| Zhejiang Jinke | POE | Slovenia | 1 |
| Zhongman Petroleum | POE | Iraq |  |
| Zhongrun Resources | POE | Mongolia | 1 |
| Zhuhai Port Holdings | SOE | Pakistan | 1 |
| Zhuzhou Kibing | POE | Malaysia | 1 |
| Zijin Mining | SOE | Kyrgyzstan | 1 |
| ZOJE Resources | POE | Russia | 1 |
| ZTE | SOE | Pakistan, Turkey | 1 |
