# Supplementary material for: The degree of internationalization of Chinese Multinationals along the belt and road initiative countries
Source: PLoS One. 2020 Jul 30;15(7):e0236636. doi: 10.1371/journal.pone.0236636 (PMC7392271; doi:10.1371/journal.pone.0236636)
Supplement: Attachment — Submitted filename: PONE-D-20-03433_reviewer1.pdf [file pone.0236636.s003.pdf]

PLOS ONE

The degree of internationalization of Chinese Multinationals along the belt and road initiative countries

--Manuscript Draft--

**Manuscript Number:**	PONE-D-20-03433
**Article Type:**	Research Article
**Full Title:**	The degree of internationalization of Chinese Multinationals along the belt and road initiative countries
**Short Title:**	The degree of internationalization of Chinese Multinationals along the belt and road initiative countries
**Corresponding Author:**	Olawoyin Gregory Adedigba, Phd Candidate<br>Nankai University Business School<br>Tianjin, Tianjin CHINA
**Keywords:**	Belt and road initiative; China; Chinese Multinationals; Degree of internationalization; State ownership; Governance: International Business; Political Economy; Regional integration; Foreign direct investment.
**Abstract:**	This study assesses the degree of internationalization(DOI) of Chinese firms along the Belt and Road initiative(BRI) countries. Most extant studies of the BRI have been qualitative, and where there have been quantitative studies they have usually been at the aggregate level and only a handful have used firm-level data to study the BRI. Using a composite measure of the degree of internationalization, we assess Chinese firms and contrary to expectations, privately owned firms had a higher DOI. Suggesting that along the belt and road countries the advantages of state ownership for Chinese MNCs is attenuated.
**Order of Authors:**	Olawoyin Gregory Adedigba, Phd Candidate
	Runhui Lin
	Nizam Ud Din
**Additional Information:**	
**Question**	**Response**
**Financial Disclosure**<br><br>Enter a financial disclosure statement that describes the sources of funding for the work included in this submission. Review the submission guidelines for detailed requirements. View published research articles from *PLOS ONE* for specific examples.<br><br>This statement is required for submission and **will appear in the published article** if the submission is accepted. Please make sure it is accurate.	The authors received no specific funding for this work.

Powered by Editorial Manager® and ProduXion Manager® from Aries Systems Corporation

**Unfunded studies**<br>Enter: *The author(s) received no specific funding for this work.*<br><br>**Funded studies**<br>Enter a statement with the following details:<br>• Initials of the authors who received each award<br>• Grant numbers awarded to each author<br>• The full name of each funder<br>• URL of each funder website<br>• Did the sponsors or funders play any role in the study design, data collection and analysis, decision to publish, or preparation of the manuscript?<br>• **NO** - Include this sentence at the end of your statement: *The funders had no role in study design, data collection and analysis, decision to publish, or preparation of the manuscript.*<br>• **YES** - Specify the role(s) played.<br><br>* typeset	
**Competing Interests**<br><br>Use the instructions below to enter a competing interest statement for this submission. On behalf of all authors, disclose any competing interests that could be perceived to bias this work—acknowledging all financial support and any other relevant financial or non-financial competing interests.<br><br>This statement **will appear in the published article** if the submission is accepted. Please make sure it is accurate. View published research articles from *PLOS ONE* for specific examples.	The authors have declared that no competing interests exist

**NO authors have competing interests**<br><br>Enter: *The authors have declared that no competing interests exist.*<br><br>**Authors with competing interests**<br><br>Enter competing interest details beginning with this statement:<br><br>*I have read the journal's policy and the authors of this manuscript have the following competing interests: [insert competing interests here]*<br><br>* typeset	
**Ethics Statement**<br><br>Enter an ethics statement for this submission. This statement is required if the study involved:<br><br>• Human participants<br>• Human specimens or tissue<br>• Vertebrate animals or cephalopods<br>• Vertebrate embryos or tissues<br>• Field research<br><br>Write "N/A" if the submission does not require an ethics statement.<br><br>General guidance is provided below. Consult the submission guidelines for detailed instructions. **Make sure that all information entered here is included in the Methods section of the manuscript.**	N/A

Powered by Editorial Manager® and ProduXion Manager® from Aries Systems Corporation

**Format for specific study types**<br><br>**Human Subject Research (involving human participants and/or tissue)**<br>• Give the name of the institutional review board or ethics committee that approved the study<br>• Include the approval number and/or a statement indicating approval of this research<br>• Indicate the form of consent obtained (written/oral) or the reason that consent was not obtained (e.g. the data were analyzed anonymously)<br><br>**Animal Research (involving vertebrate animals, embryos or tissues)**<br>• Provide the name of the Institutional Animal Care and Use Committee (IACUC) or other relevant ethics board that reviewed the study protocol, and indicate whether they approved this research or granted a formal waiver of ethical approval<br>• Include an approval number if one was obtained<br>• If the study involved *non-human primates*, add *additional details* about animal welfare and steps taken to ameliorate suffering<br>• If anesthesia, euthanasia, or any kind of animal sacrifice is part of the study, include briefly which substances and/or methods were applied<br><br>**Field Research**<br><br>Include the following details if this study involves the collection of plant, animal, or other materials from a natural setting:<br>• Field permit number<br>• Name of the institution or relevant body that granted permission	
**Data Availability**<br><br>Authors are required to make all data underlying the findings described fully available, without restriction, and from the time of publication. PLOS allows rare exceptions to address legal and ethical concerns. See the PLOS Data Policy and FAQ for detailed information.	Yes - all data are fully available without restriction

Powered by Editorial Manager® and ProduXion Manager® from Aries Systems Corporation

A Data Availability Statement describing where the data can be found is required at submission. Your answers to this question constitute the Data Availability Statement and **will be published in the article**, if accepted.<br><br>**Important:** Stating 'data available on request from the author' is not sufficient. If your data are only available upon request, select 'No' for the first question and explain your exceptional situation in the text box.<br><br>Do the authors confirm that all data underlying the findings described in their manuscript are fully available without restriction?	
**Describe where the data may be found in full sentences. If you are copying our sample text, replace any instances of XXX with the appropriate details.**<br><br>• If the data are **held or will be held in a public repository**, include URLs, accession numbers or DOIs. If this information will only be available after acceptance, indicate this by ticking the box below. For example: *All XXX files are available from the XXX database (accession number(s) XXX, XXX.).*<br>• If the data are all contained **within the manuscript and/or Supporting Information files**, enter the following: *All relevant data are within the manuscript and its Supporting Information files.*<br>• If neither of these applies but you are able to provide **details of access elsewhere**, with or without limitations, please do so. For example:<br><br>*Data cannot be shared publicly because of [XXX]. Data are available from the XXX Institutional Data Access / Ethics Committee (contact via XXX) for researchers who meet the criteria for access to confidential data.*<br><br>*The data underlying the results presented in the study are available from (include the name of the third party*	All relevant data are within the manuscript and its Supporting Information files.

and contact information or URL).<br>• This text is appropriate if the data are owned by a third party and authors do not have permission to share the data.<br><br>* typeset	
Additional data availability information:	Tick here if your circumstances are not covered by the questions above and you need the journal's help to make your data available.

Manuscript

Click here to download Manuscript Manuscript+Plos One+The DOI of Chinese MNCS Along BRI.docx

The degree of internationalization of Chinese Multinationals along the belt and road initiative countries

Woyin Adedigba, PhD student, *Nankai Business School, Nankai University, Tianjin China*; Email: *olawoyingregory@gmail.com*

Dr.Runhui Lin, Professor, *Nankai Business School, Nankai University, Tianjin China;* Email: *linrh@nankai.edu.cn*

Nizam Ud Din, PhD Student, Nankai *Business School, Nankai University, Tianjin China*; Email: *nixamuddin@yahoo.com*

Correspondence

Woyin Adedigba, PhD student, Nankai Business School, Nankai University, Tianjin China; Email: olawoyingregory@gmail.com

The degree of internationalization of Chinese Multinationals along the belt and road initiative countries

Abstract

This study assesses the degree of internationalization(DOI) of Chinese firms along the Belt and Road initiative(BRI) countries. Most extant studies of the BRI have been qualitative, and where there have been quantitative studies they have usually been at the aggregate level and only a handful have used firm-level data to study the BRI. Using a composite measure of the degree of internationalization , we assess Chinese firms and contrary to expectations, privately owned firms had a higher DOI. Suggesting that along the belt and road countries the advantages of state ownership for Chinese MNCs is attenuated.

Keywords: BRI, China, Chinese MNCs, Degree of internationalization, State ownership

1. Introduction

The one belt one road initiative (interchangeably referred to as OBOR and BRI) is an all encompassing policy initiative. It seeks to intergrate countries along the ancient silk route and the new maritime silk route, through linkages is five areas namely; policy, trade, infrastructure, finance and people [1]. This will primarily be achieved through massive infrastructure development projects.in the currently 84 countries (October,2018) that make up the BRI and since 2013 $153 Billion has so far been invested by China in BRI countries [2].

There is a positive realtionship between policy announcements and the internationalizaiton

activities of Chinese MNCs.Given China's unique institutional setting, major policy announcements have usually spawned and increased outward flows of foreign direct ineverstment[3]and policy announcements are one of the deternminants of Chinese outward FDI. In China the government enjoys significant influence on the economic landscape especially with the presence of State owned enterprises(SOES) [4–6] and uses SOEs to achieve specific policy outcomes[7].It is logical therefore, that the belt and road intitiative,the biggest and most comprehensive policy initiative yet by the Chinese government would generate a reponse by Chinese firms. Reecent studies into the effect of BRI on Chinese OFDI [4,5,8] support this.

However, dspite its economic and politiacal significance of the BRI, there are only a few empirical studies on the internationalization activities of Chinese MNCs in countries along the. The empirical studies available have focused on the magnitude and flow of foreign direct investment into the countries along the BRI. While this gives a big picture view there is room for a fine grained analysis of the BRI and Chinese firms.In this study, using the BRI as the research context, we compute the degree of internationalization of Chinese firms in the BRI countries and then assess the relationship between onwenrship type and the degree of internationalization.

The aim of this paper is to determine the degree of internationalization (DOI) of Chinese multinationals in the Belt and road countries and to assess how state ownership would affect the DOI of the Chinese multinationals along the BRI countries. The diversity of the countries comprising the belt and road initiative means that virtually all of Dunning’s[9] conditions precedent to the internationalization of firms can be found within the BRI. The BRI then essentially becomes a large pool of CSAs that Chinese firms can unlock provided they have the requisite combinatory nous to bundle them with their FSAs[10–12]. The BRI thus provides a tantalizing context to test further the relevance of extant theories on Chinese MNCS.

The degree of internationalization of chinese firms in the BRI countries DOI_{BRI} is computed using a composite index based on Sullivan's [13] DOI_{ints}. He had highlighted the drawbacks of single item measures of degree of Internationalization prevalent in the literature and proposed his multi-item index as a useful alternative. Other researchers since then[14,15] have also employed the same index or slight variations of thereof. Although, as far as we know it is yet to be utilized with Chinese firms. Comparative analysis of the DOI_{BRI} of SOEs and privately owned enterprises (POEs) turned up interesting results. Firstly, given that state ownership could be positively associated with degree of inernationalzation [16] and because of the important status of the Belt and road initiative to China we expected the SOEs to dominate the rankings in the DOI_{BRI}. Our hypothesis that because of the close involvement of the govermnent in state owned enterprises the DOI_{BRI} of the SOEs would be higher than their private counterparts, was unsupported. Lending credence to the view that under certain conditions, the behaviours of SOEs and POEs in terms of international activities converge and become indistinguishable [7,17]. Suggesting that perahaps along the BRI, the advantages bestowed by state ownership are irrelevant to successful internationaliaztion. Secondly we find that SOEs scored higher on our measure of psychic dispersion contrary to our prediction of the opposite . Furthermore, we found that POEs scored higher on the purely economic dimensions of the index.

Our research contributes both to the literature on the internationalization research in general and the internationalization of Chinese firms in particular. Also it conributes to the research into the belt and road initiative. In the next section we examine the literature and establish the theoretical foundation of this paper as well as introduce the study's hypotheses. The subsequent section covers the methodology and data analysis, after which the findings are presented. The paper concludes with a discussion of findings and implications for theory and practice.

2. Review of Literature &Theoretical Framework

2.1 Belt and road initiative research

Research into the BRI spans various academic disciplines, includingPolicy [18] Economics[19] Management and international business[4–6]. However, given that the BRI was announced and introduced in 2013, research into its influence and impact is still at infancy [5,8]. Be that as it may, scholars across disciplines acknowledge and recongise the significance and potential of the initiative.Subject matter so far investigated in the context of the BRI include inter alia the role of the RMB along the one belt one road [20,21], regional integration [22] as well as the impact of the initiative on Chinese OFDI[5,8]

One common feature of extant research is that most studies have been conductedat aggregate levels, that is, mainly at regional, national and industry level. These studies have mostly highlighted the OFDI trends along the belt and road initiative countries and only a handful of studies have examined the initiative from the firm's perspective. Also,only a few BRI studies [5,8,19,23] have been quantitative studies. This comes as a bit of a surprise considering the scope and magnitude of the initiative. The countries that make up the BRI account for more than a third of both the world's GDP (22.9 trillion USD) and population (4.6Billion) [19].

While the question of whether the BRI will bring about an increase in China's outward foreign direct investment has been answered [5,8], and there have been studies that breakdown the destinations and structure of Chinese OFDI as well, such as the excellent work of [4]. Under-investigated aspects in the literature include the actual firm level internationalization activities

Another theme in the literature is the industry distribution of the OFDI. It is evident that compared to their private counterparts SOEs domninate in the infrastructure projects along the BRI [4]. Suggesting perhaps, that SOEs are more willing to take on the significant financial burden and risks that accompany large infrastructure projects.

Furthermore, because the quantitative studies have mainly examined the causal relationship between the BRI and changes in the levels of Chinese OFDI, a preference for the Difference in differences thechnique for data analysis [4,5,8] was observed.These scholars have focused on determining the link between the launch of the BRI and changes in Chinese OFDI patterns and as there is a clear pre and post event demarcation i.e pre and post 2013, the year of BRI's launch,the technique lends itself well to such and investigation.Their findings have mainly supported a positive relationship[4,5,8].

2.2. Internationalization of Chinese firms and

In 2016, for the first time, Chinese outward FDI surpassed FDI inflows [24], becoming the second largest source of OFDI after the United States. And as China has trasnformed from a net FDI recipent to a net outward FDI destination, interest in the internationalization of Chinese firms has grown over the years [25].There is a relative abundance of research into the motives and strategies of the internationalization of Chinese multinationals [26–29] and the determinants of Chinese outward foreign direct investments include among other things market size, institutional settings and government policy [3]. While the motives for internationalization of Chinese firms are *inter alia* asset seeking, and escape from inefficient home- country institutions or "institutional arbitrage" [3,26,30–33]

The internationalization of Chinese firms (ICF) continues to generate debates in IB research as scholars are divided on the ability [29,34,35] or otherwise [36] of extant theories to explain ICF. For instance some believe that Chinese firms have by and large conformed to the predictions of the OLI paradigm [37,38] while others doubt the ability of the OLI to explain the ICF [39]. Reasons put forward for why extant theories seemingly struggle to explain Chinese MNC behaviour include neglecting the home country institutions [40], neglecting the role of local owners of complementary assets [41]

Also Rugman has argued that the successes of ICF have been blown out of proportion [29] as when compared with western MNCs in the same industry their performance lags behind. He also contends that Chinese Multinationals are regional in nature[42] and as such cannot be accurately described as engaging in internationalization.

Internationalization, loosely described is the expansion of business activities across national borders. Usually through gradual increments in their commitments in international markets [43,44]. In order to achieve this they must posses certain advantages [45]. Which according to Dunning[10,46–48] are ownership, Location and internalization advantages (OLI) or firm specific advantages(FSA) and country specific advantages(CSA) as conceived by Rugman [11,12,29,42,49]. In a nutshell the crux of the OLI framework of internationalization is that Firms begin to internationalize after they have developed sufficient capacities(ownership advantages) at home. They can then combine these with locational advantanges which can be exploited in overseas markets. The third and necessary condition for internationalization is the ability to internalize missing or ineffecient external markets[9].

For example the early internationalizing Chinese MNCS like Haier and Galanz went in pursuit of both staregeic assets and market share through their acquisitions of western firms in

the USA and France respectively while newer MNCS like Xiaomi have set their targets on gaining overseas market share, having already established a strong presence in the domestic market.

Research into ICF has been vibrant mainly because Chinese firms are relatively unique[40], studying them allows the testing of theories mainly developed with western multinationals in mind[37]. Secondly the relative speed with which Chinese multinationals have successfully internationalized flies in the face of received wisdom and has prompted calls for the development of new theories for emerging Multinationals[36], or at the very least extensions of extant theories. For instance Berning&Holtbrügge[30] assert that existing theories need to be extended or modified in order to be applicable in the Chinese context. However, others believe that extant theories have sufficient explanatory power [29,35,50]. Whether for or against extant theories, it is evident that ICF as a field of study provides a unique context for management and business research and has the potential to enrich international business and management studies.

Furthermore given that new policy announcements encourage the flow of OFDI [3,51] we can therefore expect that the launch of the BRI would precipitate an increase in OFDI and the internationalization of Chinese firms. Indeed early assessments of the BRI have found empirical support for increased OFDI resultant from the BRI [4,8]. However, as stated earlier while these studies have painted the overall picture of Chinese capital flows the individual firms and the transactions behind these flows remain unexplored. This paper aims to fill that gap.

2.3 Measuring Degree of internationalization

While the importance and relevance of internationalization to the multinational firm is intuitively clear, its measurement has remained an issue of contention in IB research[52–56]. With

questions raised over the employment of single item measures of a multidimensional concept such as internationalization[13]

There is no consensus amongst scholars concerning the choice of measurement of the degree of internationalizaiton of firms. Sullivan decried this state of affairs claiming "despite its theoretical and practical centrality, estimating the degree of internationalization (DOI) of a firm remains arbitrary" [13]. Ietto-Gillies and London echo a similar sentiment albeit less critically, stating there is no "acceptable" way to assess the degree of internationalization of firms[57], however it is still important to have a consistent measure of internationalization[52,53]. Because internationalization is multi-dimensional [57] the choice of measurement is often context specific [58].

Internationalization is a multi-dimensional concept, as such a wide array of variables can be used to operationalize its measurement[12,58].There is a location dichotomy inherent to the concept of internationalization, that is, what goes on in one location versus what goes on outside of it [59]. Generally, the home country of the multinational firm on one hand and foreign territories on the other hand.cconsequently, in developing measures for the degree of internationalization, scholars have utilized ratios that capture the percentage of foreign initiated or oriented activities to the total activities of the including the ratio of foreign sales to total sales [16,29,60] and foreign assets to total assets [61]. In addition to the location dimension mentioned earlier, other key dimensions identified in the literature include structural, attitudinal and Performance [13]; intensity, geographic- scope extensity and geographic- scope concentration [57] as well as breadth and depth scope [54] . Internationalization could be both a state and a process, a firm "is" in the former and a firm "does" in the latter. The Uppsala model [43,44] clearly emphasizes the process element of Internationalization, particularly on the gradual nature

of how firms overcome the liability of foreignness.

While some scholars have relied on the use of single item measurements, usually the ratio of foreign sales to total sales [16,29,60] others have preferred a multi item approach [14,15,52]. Although the view that composite measures are better suited to the measurement of DOI is widely held [13–15,52,59]. Ramawamy et al [62], in a critique of Sullivan [13] observed that indexed measures may not necessarily improve the understanding of internationalization. However, Sullivan [63] maintains the usefulness of a multi item index given it adequately addresses issues of construct validity and item validity. Other scholars[14,15] have utilized his DOI_{INTS} as the basis of their investigations as well.

Multi Item Indices allow for the inclusion of non-performance variables when assessing internationalization. Given that multi item measures allow for a fine grained assessment of internationalization, we follow prior scholars [13,17,52,59] in adopting a composite measure of the degree of internationalization countries.

2.4 Hypotheses

Since Chinese multinationals typically fall into two categories; state owned enterprises (SOEs), and privately owned Enterprises (POEs) and because they typically have different corporate structures, they contend with different operational constraints and the amount [64]of resources available to them. Therefore, the difference in the interactions between domestic institutions and SOEs relative to POEs as well as the resources owned by Chinese SOEs means that they usually approach internationalization differently[65]. Will the ownership structure of Chinese multinationals influence their response to BRI policy announcements and shape their internationalization efforts along the BRI countries? Or is ownership structure likely to be

irrelevant to internationalization of Chinese firms along the belt and road countries? Given that government policy is a strong determinant of Chinese OFDI[3] we expect a positive relationship between the internationalization of Chinese firms and the BRI. Furthermore, because of their unique structure, SOEs are characterized by a close working relationship with the government and because the government exerts influence on the degree of globalization of SOEs through ownership [17] and SOEs are required to fulfil national objectives[66], we propose ;

H1: SOEs would have a higher DOI_{BRI} *than POEs*

One of the principal challenges of a firm entering into international markets is how to overcome the liability of foreignness, that is, the extra costs of operating in new markets brought about by the psychic distance between the home country of the firm and the host country[12,14,43]. Psychic distance encompasses difference in culture, norms and values[67]. The BRI initiative spans more than 60 countries across continents; it stands to reason therefore that psychic distance will play a significant role in the determination of internationalization strategy. At the same time Buckley et al [3] show that geographical distance is also a significant determinant of foreign investment outflow and Du and Zhang[23] have shown that a significant percentage of OFDI into the BRI countries are to East and Central Asian countries. We conceive that because Chinese SOEs and POEs originate in the same home countries and share same cultural attributes, consequently they will contend with similar levels of liability of foreignness. We therefore propose that

H2: Along the BRI countries Psychic dispersion of SOEs and POEs would be identical

3. Materials and Methods

3.1. Sample and Data

The research employs a linear index to measure the degree of internationalization using a multi item combination. Because the decision to internationalize is inherently a firm level decision, we are interested in not just the direction and magnitude of ODI, therefore, our investigation is best served by the use of firm level data. For the use of firm level data in the analysis of Chinese OFDI provides the opportunity for more in-depth analysis[49,68]. We collected data from multiple sources, including databases including Heritage foundation's China global investment tracker, WIND and CSMAR. These three databases provide information that runs the gamut of FDI flows providing both M/A and Greenfield data. For instance the China global investment tracker provides data on all cross border deals concluded by Chinese firms worldwide that surpass the US $100 million threshold and contains data for as far back as 2005. The data includes both direct investment transactions and contracts. It also specifies which transactions are Greenfield investments. Most importantly the data distinguishes whether a recipient country belongs to the BRI or not. The use of multiple data sets allows for a triangulation of Data and therefore it improves the quality of data used in analysis.

The first step in sample generation was to extract from the global investment tracker all the transactions completed by Chinese firms from 2013 till 2018. To be included, in addition to being from 2013, it must have occurred in a BRI country. A shortlist of transactions satisfying these two conditions was compiled. The originating firms of these transactions were then identified and collated. This approach ensured that there was an even spread and allowed for a mitigation of the selection bias [3,25,29] which would have occurred had we selected the

companies from a pre-existing list, e.g. top 100 Chinese firms. There is still some form of selection bias however, as the China global investment tracker only includes transactions of a minimum of USD $100million. That means companies who have investments below that threshold will a priori not make it into our list. Global investment tracker database classifies Transactions based on whether the deal was an investment or a construction contract. Using the rubric stated above, we had 163 unique firms who had been involved in investment transactions and 94 firms who primarily had construction transactions giving a total of 257 firms. The shortlist is summarized in table 1 below.

[Insert table 1 here]

In the second stage only firms with publicly available data were retained. These were composed primarily of listed firms and firms like Huawei that although not listed have publically available data. This was necessitated by data availability challenges, as listed firms have disclosure requirements therefore the necessary data is readily available. The requisite data on the short-listed firms were then sourced from the WIND database as well CSMAR in addition to the published annual results. These include the foreign and total sales, overseas and foreign subsidiaries.

Finally we applied the constraint that firms must have significant operations or activities in overseas markets. Internationalization as a concept presupposes the initiation of and participation in cross border business activities, in any case Rugman et al [29] defined the multinational firm as a firm with at least ten percent of sales in foreign markets and with at least three foreign subsidiaries. The multinational firm is also one that engages in value addition activities across borders[9] as well as one that organizes the linkages between employees located in more than one country through employment contracts [39], while Fitzgerald & Rowley[69] define it as a

firm that has significant investments and large scale business activities in different countries, while maintaining ownership and control. Considering these attributes of MNCS, our definition isn't as weighted towards the proportion of foreign sales as an indicator of multi-nationality *a la* Rugman. For instance ALIBABA had no foreign sales for the period under review. Yet it had 30 foreign subsidiaries, which indicates a significant investment and involvement in overseas markets albeit one suggestive of a strategic rather than commercial nature. Were we to apply the 10% of total sales condition we would have had to exclude Alibaba from the sample which would be odd given the evidence of its international involvement. This underscores Sullivan's [13] point that the use foreign sales ratio is not a sufficient measure of internationalization. In any case the motivations for Chinese firms to internationalize vary, from the purely market seeking to the strategic asset seeking motive[9] to the strategic intent perspective [70]. Therefore to be included in our sample firms must have engaged in FDI along the BRI countries in addition to either having a foreign subsidiary and or have sales from overseas markets. This conceptualization of multi-nationality is broadly in agreement with Dunning's definition of the multinational as "an enterprise that engages in foreign direct investment (FDI) and owns or, in some way, controls value-added activities in more than one country"[9]

With this final criterion, our sample was reduced to 92 firms. A major casualty of this stage was JD the Chinese e-commerce giant, with neither foreign sales nor foreign subsidiaries.[1]

[1]One possible explanation for this is that while the global investment tracker captures JD's new investment along the BRI the Historical data which we used in sample selection will not contain the records of Its investments. This should be one of the limitations of our study

3.2 Variables

Owing to both the plurality of the conceptualization of internationalization and its multidimensionality, there are several approaches to the measurement of the degree of internationalization of firms

Sullivan's [13] DOI_{INTS} was a 5 item composite index made up of a linear combination of ratios. The ratios were FS/TS foreign sales and total sales, FA/TA foreign assets and total assets, TMIE top managers' international experience and psychic dispersion of operations. We use a modified version of the $DOI_{INTS,}$ for this study. Owing to data availability constraints, the foreign asset to total assets ratio was omitted as was the TMIE, while including a measure for geographical dispersion a la [59]. We use a four item index as shown below. The DOI is measured for each shortlisted firm by plugging the ratios for each firm into the equation below. In adopting this approach, we are in agreement with Dörrenbächer[71] that Sullivan's measure is one of the few that combines the main dimensions of Internationalization into one measure.

$$DOI_{=}FSTS + OSTS + PDIO + GD \quad (1)$$

The main dimensions of internationalization indentified in the literature include; performance, structural and attitudinal dimensions [13,71], intensity and extensity dimensions [59] real and financial [58]. We do find however that the dimensions and the variables to operationalize them often overlap. For instance while Sullivan[13] considers foreign sales to total sales ratio as a performance variable, Ietto-Gilles [59] considers it a measure of intensity, the value of activities per location. This difference in our view is semantic, as they still capture the same effect. Consequently we ensured key dimensions of internationalization were captured by our measure. For instance, the omission of the assets ratio, which expressed the structural

dimension in Sullivan[13] does not significantly impact the usefulness of the index as the structural dimension is still aptly captured by the subsidiary ratio. While the sales ratio still captures the performance dimension and psychic dispersion operationalizes the attitudinal dimension. In essence we have one ratio to capture each dimension as identified by Sullivan[13]. The average of the 3 years 2015,2016 ,2017 were used to compute FSTS , while the OSTS value was determined using single year data in this case year 2017.

3.2.1 Performance/Intensity (FSTS)

This is the most used measure of the DOI [49]. Many studies have solely utilized this variable to measure the degree of internationalization[60,72]. While it is indeed a good proxy, it has flaws because it is inherently biased towards large firms and firms with an inordinate amount of foreign sales that might originate in a single market for instance[53]. And as observed by Ietto-Gillies and London [57] the foreign sales to total sales ratio mainly focuses on the home vs foreign dichotomy without providing insights dispersion. While it adequately captures the performance dimension of internationalization, it fails to capture the other dimensions of internationalization. For example if we take two multi-national companies X and Y, X with foreign sales of $10million and Y with $5 million, where X's foreign sales come from two countries, and Y from 5 territories. If both had total sales of $20 million and we compute their foreign sales to total sales ratio, that measure will show X as having a higher degree of internationalization. This would completely ignore the associated complexity with operating in more geographically diverse and potentially psychically diverse markets. This is why the FSTS alone does not paint a robust picture of the internationalization activities of firms.

FSTS captures the quantum of business done by the MNC and what proportion of its

performance comes from international markets. It is a good first order measure of international involvement of the firm. If a firm is a strategic asset seeking international firm, the FSTS measure may not adequately capture this aspect of internationalization. We argue that it focuses too narrowly on the market seeking motive of internationalization; therefore it is better to use it as part of a multi variable index rather than a stand-alone measure.

3.2.2 Structure (OSTS)

Following Sullivan[13,63] we use OSTS to capture the structural dimension of internationalization. Since Chinese firms also base their internationalization on rational analysis[70]the types and locations of Chinese MNCs subsidiaries may reflect their strategic intent given subsidiaries vary in role and type[73] e.g overseas subsidiaries that are not directly involved in revenue generating activities may focus on R&D, administration or even be the so called centre of excellence [74].Subsidiaries located in tax havens may contribute immensely to the bottom line of the firm even though they might not generate revenues from sales. And as highlighted by Hassel et al [58] their investigation of financial internationalization of German firms conclude that extant studies often focused the production or real dimension of internationalization to the exclusion of governance dimensions of internationalization. We acknowledge the non financial dimensions of internationalization by considering OSTS not just as a purely structural measure but as well as an indicator of strategic intent.

3.2.3 Psychic Dispersion (PDIO)

The liability of foreignness MNCS face is a key determinant of the success of their internationalization process [12,44,75] and psychic distance influences the liability of foreignness [43]. The variable PDIO measures the psychic dispersion of the firm’s operations.

That is, how psychically diverse firm is along the belt and road initiative countries. Sullivan used Ronen and Shenkar's[76] psychic maps to measure the psychic dispersion. As it is possible to have territories which might be geographically proximate but psychically or culturally distant and vice versa where countries are psychically proximate in spite of geographical distance it is useful to measure the psychic dispersion. An easy example would be Great Britain and the USA's cultural proximity even though they are separated by a large geographical distance.

Although, Sousa & Bradley [67] make a distinction between psychic and cultural distance we find either conceptualization sufficient for our study because they both relate to how distinctive attitudinal traits compare and vary across locations. Having said that however, it became quickly apparent that Ronen and Shenkar's[76] classification would not suffice for this study. Ramaswamy et al[62] already pointed out that the country clusters did not sufficiently address the diversity in the world, we share their view that the 'independent' group does not really provide any useful insight. For example using this study's sample of the belt and road countries, the Ronen and Shenkar[76] Classification classifies both Nigeria and Croatia as "independent" , and one would be hard pressed to argue that these two countries have similar psychic make ups. The Global investment tracker's classification also proved inadequate, as their classification is more geographically oriented rather than psychological or cultural. We modified Ronen and Shenkar's[76] classification to better suit the study by including a tenth cluster– Sub-Saharan Africa. We find this more relevant to the study as a significant number of belt and road countries are found here, moreover there's more to be said for the psychic similarity of Nigeria and Cameroon than for Nigeria's and Croatia. Table 2 shows the classification.

[Insert table 2 here]

3.2.4 Geographic dispersion

The uniqueness of the BRI is that it encompasses many countries, therefore it is useful to identify how widespread the activities of Chinese multinationals are, along the belt and road initiative. Consequently, a fourth item was included in our index, it is intended to capture the Geographic extensity of the firms along the Belt and road initiative countries, given the relevance of geographic diversity[77] in this context. We follow Ietto-Gilles [59] in using count of the number of countries along the belt and road initiative countries where the firm had activities, normalised by the total number of belt and road countries which stood at 84 at the time of this paper. Furthermore, Ramaswamy et al [62] argued in fact that a simple count of the number of countries may be a better measure of the dispersion of internationalization of Multinational firms, and this study adopts it as our measure of geographical dispersion. Below, Table.3 presents a description of the variables and data sources.

[Insert table 3 here]

FSTS and OSTS correspond to both the intensity dimension of Ietto-Gilles and performance dimensions of Sullivan. PDIO captures the attitudinal dimension as identified by Sullivan. While the last variable, GD pertains to the geographical extensity identified in Ietto- Giles[59].

4. Results

The average DOI_{BRI} of the sample was 0.67 and when broken down into SOEs and POEs we have 0.59 and 0.75 respectively Table.4 presents the descriptive statistics showing the POEs vs SOEs and Table 5 overall descriptive statistics. Indicating a higher DOI_{BRI} for privately owned enterprises compared to state owned enterprises. As a matter of fact, only two SOEs were in the top ten as ranked by the DOI_{BRI} values. Qingjjian a POE recorded the highest DOI_{BRI} value.

Consequently, H_1 is unsupported and rejected. On the psychic dispersion measure, SOEs had an average of 0.18 while for POEs the value was 0.11, indicating that the SOES scored higher on that measure therefore H_2 is unsupported. If we looked at the dispersion measures alone, the SOEs indeed ranked higher than the POEs, this suggests that perhaps SOES are unfazed by and better equipped to deal with the challenges of operating in more psychically and geographically dispersed locations. It may also be that state ownership has less influence on the internationalization of Chinese firms along the Belt and road initiative and other moderator variables are better associated. [78], for instance, found market potential to be a strong determinant of Chinese OFDI along the Belt and Road countries.

[Insert table 4 here]

[Insert table 5 here]

The rankings of the other dimensions revealed that SOEs ranked higher in the extensity dimensions, That is, the PDIO and dispersion measure, whereas the POEs ranked higher in the intensity and performance measures, FSTS and OSTS. Overall, even though the number of firms in the sample was almost identical between SOEs and POEs (47 and 44 respetively) , we discovered that the firms with the highest DOIs were POES and not SOEs. This was unexpected as given the relative financial might of SOEs and typical preferential treatment enjoyed by SOEs the expectation was that they would be at the vanguard of the internantionalization rankings along the belt and road coutnries. It should be noted however, that in terms of pure numbers of transactions initiated, the SOEs still rule the roost. It is only when this is normalized do we see the result discussed above. This is perhaps illustrative of the usefulness of a multi item index measure of DOI, as it discounts firm size . We also found that Alibaba has a preponderance of its subsidiaries abroad, with approximately 70% of its total subsidiaries located overseas. This

however contrasts with its foreign to total sales ratio where no figures for foreign sales were recorded in the annual reports

The correlation matrix presented in Table.6 indicates a strong and positive correlation between the dispersion variables PDIO and GD at 0.85. Cronbach's alpha of the index was 0.4889. These measures are however negatively correlated with the both FSTS and OSTS. This is in line with the prediction of Uppsala model (Johanson & Vahlne, 1977, 2009) that firms would expand into psychically and geographically proximate locations before venturing further afield.

[Insert table 6 here]

5. Discussion

Data availailibilty has remained a major bane to the measuremnent of DOI [71] and as articulated by several scholars[13,52,71,77,80] researchers are contrained by available and accessible data. This study suffers the same fate, as we had to omit certain variables owing to data unavilability/inaccessibilty,for example the top management international experience. While we acknowledge that alternative methods or data may exist , our study was carried our within this constraint.

As regards items comprising the index, computed Cronbach's alpha of 0.48 barely lies within acceptable limits of 0.7 for exploratory research that is widely cited in the literature[13,15]. However the usefulness of the alpha in measuing validity and reliability has been questinoned [81] because Cronbach's alpha is inlfuenced by factors that may not reflect the reliablity of a scale [82] as well as by the number of items in the scale and the sample size, our limited sample size is the likely source of the low value rather than the unsuitability of the

measure. In any case Panayides[83], advises caustion when reposrting alpha values as higher values do not necessarily indicate higher reliability.And ex ante, aplha is neither capable of measuring unidimensionanlity nor reliability[84]. Given that the goal of DOI_{bri} was to capture the multidimensional nature of internationalization and higher alpha values could be associated with a narrow coverage of a construct [83] our alpha value does not debase the utility of the index

Furthermore, our finding that the SOEs did not have a higher DOI as predicted could be down to the fact that given the development of Chinese MNCs over the last decade , POEs have been able to amasss fromidable resources and developed capabilities that neutralize the advantages accrued by SOEs . It is also possible that because the advantages enjoyed by SOEs were borne of the relatively weak institutions and market inefficiencies characteristic of China and because there's a relationship betweeen reforms and DOI [17], recent reforms have been successful to such an extent that they have degraded the advantages of SOEs over POEs. Although the instiutional arbitrage[85] perspective is that POEs may be escapting disadvantageous positions in the home market, the fact that there isn't such a wide variance between the mean DOI of the SOEs and POEs, suggests that at least within the BRI context, this may no be the case. However, these explanations may only be releavant in the context of the BRI because being the brainchld of the government, deliberate measures may have been taken to reduce the red tape that would have adverserly affected iternationalization efforts of POEs. Future research could test this by comparing the activies of POEs in BRI to non BRI countries.

In testing our second hypothesis, we found SOEs had a higher PDIO score which was indicative of more psychic dispersion. It is possible that SOEs are better equipped to deal with the liabilities of foreignness that goes with operating in psychically distant countries. It could be

for instance that since SOEs usually prefer government to government relationships [64] they are better able to get host countries' governments to safeguard their investments which in turn means mitigates the liability of foreignness. The fact that SOEs have a preponderance of their activities in the infrastructure development projects in the BRI countries[4], lends credence to this, as these projects are likely to be negotiated, agreed and commissioned at the highest levels of host country governments. This suggests that while state ownership may not matter much to the overall degree of internationalization, but that it is important to overcoming liability of foreignness.

Firm Age and stage may moderate the degree of internationalization [53] and given that some of the POEs enjoyed a perfect OSTS score, our findings appear to support this. As some of them have only one subsidiary which happens to be the foreign subsidiary, which suggests they are likely at an early stage of the internationalization.When compared to the more estbablished firms with a longer history of internationalization,who may already have shifted focus in other directions or regions, it may be that the BRI provides the appropriate platforms for newly internationalizing firms to expand their foreign opeations. And with the overarching goal of the belt and road intitiative being integration in the stated areas of policy, trade, infrastructure, finance and people[1], it is possible that these efforts are already bearing fruits and the internationalization process along the BRI countries may have become easier owing to the commitment of the various national governments to see it succeed.

Also, there is *prima facie* evidence that Aliababa's overseas subsidiaries may not be revenue generating. A possible explanation would be that they serve a strategic rather than commercial purpose, e.g as R&D centres or as administrative hubs. It seems a logical explanation Given the primacy of logistics in the Alibaba business model. Consequently, a

thorough study of these subsidiaries may provide addditional insights.

Following from above, since firm governance influences interantional behaviour [86] and there are different governance regimes that affect SOES [17] while strict corporate governance requirements negate state ownership [87] because our sample consists of listed firms, both POEs and SOEs would be subject to the same governance requirements. In any case, Estrin et al[61] posit that listed SOEs are in fact Hybrid firms, and they may be closer in outlook to POEs than to non listed SOES. Future research could compare the internationalization of listed and non listed SOEs

Lastly, this study is only the first attempt to compute the DOI of Chinese firms in the context of the BRI, and while an attempt has been made to us\tiliize as robust a measeure as possible, there are still avenues to improve the methodological approach. For instance using the complex number developed by Fisch and Oesterle[52], may provide an alternative quantitative measure that allows meaningful comparisons to be made .

6. Conclusion

Given the scope and magnitude of the belt and road initiative, and the unique context it presents for research into the internationalization of Chinese firms, this study attempted to compute the degree of internationalization of Chinese multinationals along the belt and road initiative countries. Using a composite measure, a multi-item index based on Sullivan [13], Chinese multinationals who had initiated transactions in Belt and road countries in the period 2013-2018 were compiled from the global investment tracker data base. A bottom up approach was preferred as the transactions, were first identified before unmasking the companies responsible for the transactions.

Even though there was no “right” way to measure the degree of internationalization found in the literature, a multi item indexed was preferred over a single measure of the degree of internationalization given the superiority of multi item measures of DOI .The index used, comprised of four items, FSTS, OSTS, PDIO and Geographic Dispersion, which measured the ratio of foreign sales to total sales, overseas subsidiaries to total subsidiaries , psychic dispersion and geographic dispersion respectively. It was hypothesized that state owned enterprises would rank higher than privately owned enterprises and that psychic distance would influence SOES and POEs equally through an identical level of psychic dispersion within the BRI countries. Both *H1* and *H2* were unsupported and consequently rejected. Privately owned enterprises indeed had a higher averge DOI than the State owned enterprises and SOES scoered higher on the PDIO measurre, indicating on average a psychically diverse operation. This result suggests that perhaps the internationalization of Chinese firms has matured tosuch a stage that the effects of ownership structure vis a vis state ownership is approaching insignificance. This corporate maturity would imply that one of the main explanations often cited as the reason for the special nature of the internatioalizaition of Chinese firms may have dissipated in relevance, Chinese firms may not now be that much different compared to their western counterparts and consequently extant theories may do a better Job of explainng their behaviour. This notion ought to be tested in future research. Although it should be added that the determinants of Chinese investments in BRI differ to their determinants in non BRI countries [78]

Inspite of the limitatinons of this study, it contributes to the literature on the intenationaliztion of Chinese firms as well as the belt and road initiative. It is a first order level for assessing the impact of the policy. High DOI along the BRI would indicate the acceaptance of the policy although with the caveat that some of the investments and projects may have been

planned earlier. Prima facie, the BRI as policy seems to have spurred internationalization equally between SOEs and POEs

References

1. Chin H, He W. The Belt and Road Initiative: 65 Countries and Beyond. Fung Business Intelligence Centre. 2016.

2. Perez C, Scissors D. A close look at OBOR reveals overstated gains. In: AEI [Internet]. 16 May 2017 [cited 16 Apr 2019]. Available: http://www.aei.org/publication/a-close-look-at-obor-reveals-overstated-gains/
3. Buckley PJ, Clegg LJ, Cross A, Liu X, Voss H, Zheng P. The determinants of Chinese outward foreign direct investment. Foreign Direct Investment, China and the World Economy. Springer; 2010. pp. 81–118.
4. Du J, Zhang Y. Does One Belt One Road initiative promote Chinese overseas direct investment? China Economic Review. 2018;47: 189–205. doi:10.1016/j.chieco.2017.05.010
5. Lv P, Guo C, Chen X. How the Belt and Road Initiative Affects China's Outward FDI: Comparing Chinese Independent Firms and Business Group Affiliates. In: Zhang W, Alon I, Lattemann C, editors. China's Belt and Road Initiative. Cham: Springer International Publishing; 2018. pp. 243–263. doi:10.1007/978-3-319-75435-2_13
6. Zhai F. China's belt and road initiative: A preliminary quantitative assessment. Journal of Asian Economics. 2018;55: 84–92. doi:10.1016/j.asieco.2017.12.006
7. Estrin S, Meyer KE, Nielsen BB, Nielsen S. Home country institutions and the internationalization of state owned enterprises: A cross-country analysis. Journal of World Business. 2016;51: 294–307. doi:10.1016/j.jwb.2015.11.002
8. Kang L, Peng F, Zhu Y, Pan A. Harmony in Diversity: Can the One Belt One Road Initiative Promote China's Outward Foreign Direct Investment? Sustainability. 2018;10: 3264. doi:10.3390/su10093264
9. Dunning JH, Lundan SM. Multinational enterprises and the global economy. 2nd ed. Cheltenham, UK ; Northampton, MA: Edward Elgar; 2008.
10. Dunning JH. The Eclectic (OLI) Paradigm of International Production: Past, Present and Future. International Journal of the Economics of Business. 2001;8: 173–190. doi:10.1080/13571510110051441
11. Rugman AM. Reconciling Internalization Theory and the Eclectic Paradigm. Multinational Business Review. 2010;18: 1–12. doi:10.1108/1525383X201000007
12. Rugman AM, Verbeke A, Nguyen QTK. Fifty Years of International Business Theory and Beyond. Management International Review. 2011;51: 755–786. doi:10.1007/s11575-011-0102-3
13. Sullivan D. Measuring the degree of internationalization of a firm. Journal of

international business studies. 1994;25: 325–342.
14. Pangarkar N. Internationalization and performance of small- and medium-sized enterprises. Journal of World Business. 2008;43: 475–485. doi:10.1016/j.jwb.2007.11.009
15. Reuber AR, Fischer E. The influence of the management team's international experience on the internationalization behaviors of SMEs. Journal of International Business Studies. 1997;28: 807–825.
16. Hobdari B, Gregoric A, Sinani E. The role of firm ownership on internationalization: evidence from two transition economies. J Manag Gov. 2011;15: 393–413. doi:10.1007/s10997-009-9112-y
17. Liang H, Ren B, Sun SL. An anatomy of state control in the globalization of state-owned enterprises. Journal of International Business Studies. 2015;46: 223–240. doi:10.1057/jibs.2014.35
18. Zhang J, Wu Z. Effects of Trade Facilitation Measures on Trade Between China and Countries Along the Belt and Road Initiative. In: Zhang W, Alon I, Lattemann C, editors. China's Belt and Road Initiative. Cham: Springer International Publishing; 2018. pp. 227–241. doi:10.1007/978-3-319-75435-2_12
19. Hu B, Pan Q, Wu S. The Overall Development of the Belt and Road Countries: Measurement, Ranking, and Assessment. In: Zhang W, Alon I, Lattemann C, editors. China's Belt and Road Initiative. Cham: Springer International Publishing; 2018. pp. 201–225. doi:10.1007/978-3-319-75435-2_11
20. Arase D. China's two silk roads initiative: What it means for Southeast Asia. Southeast Asian Affairs. 2015;2015: 25–45.
21. Jianqiu Y, Zhe C. The analysis and forecast of RMB internationalization on one belt and one road. International Business and Management. 2015;10: 137–141.
22. Summers T. China's 'New Silk Roads': sub-national regions and networks of global political economy. Third World Quarterly. 2016;37: 1628–1643. doi:10.1080/01436597.2016.1153415
23. Du J, Zhang Y. Does One Belt One Road initiative promote Chinese overseas direct investment? China Economic Review. 2017 [cited 12 Jan 2018]. doi:10.1016/j.chieco.2017.05.010
24. UNCTADstat. [cited 27 Nov 2018]. Available: http://unctadstat.unctad.org/EN/Index.html
25. Deng P. Chinese Outward Direct Investment Research: Theoretical Integration and Recommendations. Management and Organization Review. 2013;9: 513–539. doi:10.1111/more.12030
26. Child J, Rodrigues SB. The Internationalization of Chinese Firms: A Case for Theoretical Extension? Management and Organization Review. 2005;1: 381–410. doi:10.1111/j.1740-8784.2005.0020a.x
27. Deng P. Why do Chinese firms tend to acquire strategic assets in international expansion? Journal of World Business. 2009;44: 74–84. doi:10.1016/j.jwb.2008.03.014
28. Ramamurti R, Hillemann J. What is "Chinese" about Chinese multinationals? Journal of International Business Studies. 2017. doi:10.1057/s41267-017-0128-2
29. Rugman AM, Nguyen QTK, Wei Z. Rethinking the Literature on the Performance of Chinese Multinational Enterprises. Management and Organization Review. 2016;12: 269–302. doi:10.1017/mor.2016.13

30. Berning SC, Holtbrügge D. Chinese outward foreign direct investment—a challenge for traditional internationalization theories? Journal für Betriebswirtschaft. 2012;62: 169–224.
31. Deng P. Chinese Outward Direct Investment Research: Theoretical Integration and Recommendations. Management and Organization Review. 2013;9: 513–539. doi:10.1111/more.12030
32. Guo J, Wang G, Tung C. Do China's Outward Direct Investors Prefer Countries with High Political Risk? An International and Empirical Comparison. China & World Economy. 2014;22: 22–43. doi:10.1111/cwe.12090
33. Meyer MW. Going Out by Going In: Business Model Innovation with Chinese Characteristics: Going Out by Going In. Thunderbird International Business Review. 2017;59: 473–482. doi:10.1002/tie.21893
34. Benito GR. Why and how motives (still) matter. The Multinational Business Review. 2015;23: 15–24.
35. Guillén MF, García-Canal E. The American model of the multinational firm and the "new" multinationals from emerging economies. Academy of Management Perspectives. 2009;23: 23–35.
36. Hemmert M, Jackson K. Is there an East Asian model of MNC internationalization? A comparative analysis of Japanese and Korean firms. Asia Pacific Business Review. 2016;22: 567–594. doi:10.1080/13602381.2016.1168617
37. Erdener C, Shapiro DM. The Internationalization of Chinese Family Enterprises and Dunning's Eclectic MNE Paradigm. Manag Organ Rev. 2005;1: 411–436. doi:10.1111/j.1740-8784.2005.00021.x
38. Jason Li-Ying, Tamara Stucchi, Anne Visholm, Joanna Solvig Jansen. Chinese multinationals in Denmark: Testing the eclectic framework and internalization theory. Multinational Business Review. 2013;21: 65–86. doi:10.1108/15253831311309492
39. Hennart J-F. Down with MNE-centric theories! Market entry and expansion as the bundling of MNE and local assets. Journal of International Business Studies. 2009;40: 1432–1454.
40. Peng MW. The global strategy of emerging multinationals from China. Global Strategy Journal. 2012;2: 97–107.
41. Hennart J. Emerging market multinationals and the theory of the multinational enterprise. Global Strategy Journal. 2012;2: 168–187.
42. Collinson S, Rugman AM. The regional character of Asian multinational enterprises. Asia Pacific Journal of Management. 2007;24: 429–446. doi:10.1007/s10490-006-9035-y
43. Johanson J, Vahlne J-E. The Internationalization Process of the Firm-A Model of Knowledge Development and Increasing Foreign Market Commitments. Journal of International Business Studies. 1977;8: 23–32.
44. Johanson J, Vahlne J-E. The Uppsala internationalization process model revisited: From liability of foreignness to liability of outsidership. Journal of International Business Studies. 2009;40: 1411–1431. doi:10.1057/jibs.2009.24
45. Hymer SH. International operations of national firms. 1976.
46. Dunning JH. Toward an Eclectic Theory of International Production: Some Empirical Tests. J Int Bus Stud. 1980;11: 9–31. doi:10.1057/palgrave.jibs.8490593
47. Dunning JH. The theory of international production. The International Trade Journal. 1988;3: 21–66. doi:10.1080/08853908808523656

48. Dunning JH. The Eclectic Paradigm of International Production: A Restatement and Some Possible Extensions. In: Cantwell J, editor. The Eclectic Paradigm. London: Palgrave Macmillan UK; 2015. pp. 50–84. doi:10.1007/978-1-137-54471-1_3
49. Rugman AM, Verbeke A. Liabilities of regional foreignness and the use of firm-level versus country-level data: a response to Dunning et al. (2007). Journal of International Business Studies. 2007;38: 200–205. doi:10.1057/palgrave.jibs.8400242
50. Wei Z. The literature on Chinese outward FDI. Multinational Business Review. 2010;18: 73–112.
51. Luo Y, Xue Q, Han B. How emerging market governments promote outward FDI: Experience from China. Journal of World Business. 2010;45: 68–79. doi:10.1016/j.jwb.2009.04.003
52. Fisch JH, Oesterle M-J. Exploring the globalization of German MNCs with the complex spread and diversity measure. Schmalenbach Business Review. 2003;55: 2–21.
53. Glaum M, Oesterle M-J. 40 years of research on internationalization and firm performance: more questions than answers? 2007.
54. Marano V, Arregle J-L, Hitt MA, Spadafora E, van Essen M. Home Country Institutions and the Internationalization-Performance Relationship: A Meta-Analytic Review. Journal of Management. 2016;42: 1075–1110. doi:10.1177/0149206315624963
55. Nguyen QT. Multinationality and performance literature: A critical review and future research agenda. Management International Review. 2017;57: 311–347.
56. Nguyen T-H, Cosset J-C. Applied Economics. 1995;27: 343–351. doi:10.1080/00036849500000118
57. Ietto-Gillies G, London S. Conceptual issues behind the assessment of the degree of internationalization. Transnational Corporations. 2009;18: 59–83.
58. Hassel A, Höpner M, Kurdelbusch A, Rehder B, Zugehör R. Two Dimensions of the Internationalization of Firms*. Journal of Management Studies. 2003;40: 705–723. doi:10.1111/1467-6486.00357
59. Ietto-Gillies G. Different conceptual frameworks for the assessment of the degree of internationalization: an empirical analysis of various indices for the top 100 transnational corporations. Transnational corporations. 1998;7: 17–40.
60. Ren S, Eisingerich AB, Tsai H-T. How do marketing, research and development capabilities, and degree of internationalization synergistically affect the innovation performance of small and medium-sized enterprises (SMEs)? A panel data study of Chinese SMEs. International Business Review. 2015;24: 642–651.
61. Estrin S, Meyer KE, Nielsen BB, Nielsen S. Home country institutions and the internationalization of state owned enterprises: A cross-country analysis. Journal of World Business. 2016;51: 294–307. doi:10.1016/j.jwb.2015.11.002
62. Ietto-Gillies G. Different conceptual frameworks for the assessment of the degree of internationalization: an empirical analysis of various indices for the top 100 transnational corporations. Transnational corporations. 1998;7: 17–40.
63. Ramaswamy K, Kroeck KG, Renforth W. Measuring the degree of internationalization of a firm: A comment. Journal of International Business Studies. 1996;27: 167–177.
64. Sullivan D. Measuring the degree of internationalization of a firm: A reply. Journal of international business studies. 1996;27: 179–192.
65. Ramasamy B, Yeung M, Laforet S. China's outward foreign direct investment: Location choice and firm ownership. Journal of world business. 2012;47: 17–25.

66. Huang X, Renyong C. Chinese Private Firms? Outward Foreign Direct Investment: Does Firm Ownership and Size Matter? Thunderbird International Business Review. 2014;56: 393–406. doi:10.1002/tie.21629
67. Wei Z, Nguyen QTK. Subsidiary strategy of emerging market multinationals: A home country institutional perspective. International Business Review. 2017;26: 1009–1021. doi:10.1016/j.ibusrev.2017.03.007
68. Sousa CMP, Bradley F. Cultural distance and psychic distance: refinements in conceptualisation and measurement. Journal of Marketing Management. 2008;24: 467–488. doi:10.1362/026725708X325959
69. Amighini A, Cozza C, Rabellotti R, Sanfilippo M. Investigating Chinese Outward Foreign Direct Investments: How Can Firm-level Data Help? China & World Economy. 2014;22: 44–63. doi:10.1111/cwe.12091
70. Fitzgerald R, Rowley C. Japanese multinationals in the post-bubble era: new challenges and evolving capabilities. Asia Pacific Business Review. 2015;21: 279–294.
71. Rui H, Yip GS. Foreign acquisitions by Chinese firms: A strategic intent perspective. Journal of World Business. 2008;43: 213–226.
72. Dörrenbächer C. Measuring corporate internationalisation: A review of measurement concepts and their use. Intereconomics. 2000;35: 119–126. doi:10.1007/BF02927197
73. Filatotchev I, Piesse J. R&D, internationalization and growth of newly listed firms: European evidence. Journal of International Business Studies. 2009;40: 1260–1276.
74. Birkinshaw J, Hood N, Jonsson S. Building firm-specific advantages in multinational corporations: the role of subsidiary initiative. Strategic Management Journal. 1998;19: 221–242. doi:10.1002/(SICI)1097-0266(199803)19:3<221::AID-SMJ948>3.0.CO;2-P
75. Enright MJ, Subramanian V. An organizing framework for MNC subsidiary typologies. Management International Review. 2007;47: 895–924. doi:10.1007/s11575-007-0057-6
76. Casillas JC, Acedo FJ. Speed in the Internationalization Process of the Firm. International Journal of Management Reviews. 2013;15: 15–29. doi:10.1111/j.1468-2370.2012.00331.x
77. Ronen S, Shenkar O. Clustering Countries on Attitudinal Dimensions: A Review and Synthesis. Academy of Management Review. 1985;10: 435–454. doi:10.5465/amr.1985.4278955
78. Liu HY, Tang YK, Chen XL, Poznanska J. The Determinants of Chinese Outward FDI in Countries Along "One Belt One Road." Emerging Markets Finance and Trade. 2017;53: 1374–1387. doi:10.1080/1540496X.2017.1295843
79. Johanson J, Vahlne J-E. The internationalization process of the firm—a model of knowledge development and increasing foreign market commitments. Journal of international business studies. 1977;8: 23–32.
80. Ietto-Gillies G, London S. Conceptual issues behind the assessment of the degree of internationalization. Transnational Corporations. 2009;18: 59–83.
81. Sullivan D. Measuring the Degree of Internationalization of a firm: A Reply. Journal of International Business Studies. 1996;27: 179–192. doi:10.1057/palgrave.jibs.8490132
82. Sijtsma K. On the Use, the Misuse, and the Very Limited Usefulness of Cronbach's Alpha. Psychometrika. 2009;74: 107–120. doi:10.1007/s11336-008-9101-0
83. Shevlin M, Miles J, Davies M, Walker S. Coefficient alpha: a useful indicator of reliability? Personality and individual differences. 2000;28: 229–237.
84. Panayides P. Coefficient alpha: interpret with caution. 2013.

85. Peters G-J. The alpha and the omega of scale reliability and validity: why and how to abandon Cronbach's alpha and the route towards more comprehensive assessment of scale quality. 2014.
86. Boisot M, Meyer MW. Which way through the open door? Reflections on the internationalization of Chinese firms. Management and Organization Review. 2008;4: 349–365.
87. Gaur A, Delios A. International Diversification of Emerging Market Firms: The Role of Ownership Structure and Group Affiliation. Manag Int Rev. 2015;55: 235–253. doi:10.1007/s11575-015-0240-0
88. Arreola F, Bandeira-de-Mello R. The Differential Effects of Minority State Ownership Types on the Internationalization of Emerging Market Multinationals from Democratic States. Manag Int Rev. 2018;58: 845–869. doi:10.1007/s11575-018-0352-4

Tables

Table 1 Chinese deals along BRI countries from 2013-2018

Item	Investment	Contracts
No of deals 2013-2018	248	527
No of initiating companies	163	94
Dollar value	$153 billion	$267 billion
No of countries	46	57
Regions	6	7

Source: China global investment tracker

Table 2. The countries of the BRI mapped on a modified version of Ronen and Shenkar's Psychic Zones that includes Sub-Saharan Africa

Nordic	Germanic	Anglo	Latin Europe	Latin America	Far Eastern	Arab	Near Eastern	Independent	Sub Saharan
	Austria	New Zealand	Portugal	Bolivia	Bangladesh	Algeria	Afghanistan	Antigua and Barbuda	Angola
				Chile	Brunei	Bahrain	Albania	Dominica	Burundi

	Costa Rica	Cambodia	Egypt	Azerbaijan	Fiji	Cameroon
	Dominican Republic	India	Iraq	Belarus	Grenada	Cape Verde
	El Salvador	Indonesia	Jordan	Bhutan	Guyana	Chad
	Uruguay	Laos	Kuwait	Bosnia and	Herzegovina	Congo
	Venezuela	Malaysia	Lebanon	Bulgaria	Hungary	Cote d'Ivoire
		Myanmar	Libya	Croatia	Israel	Djibouti
		Nepal	Morocco	Czech Republic	Lithuania	Ethiopia
		Pakistan	Oman	Estonia	Malta	Gabon
		Philippines	Qatar	Georgia	Mauritania	Gambia
		Republic of Korea	Saudi Arabia	Greece	Moldova	Ghana
		Singapore	Syria	Iran	Mongolia	Guinea
		Sri Lanka	Tunisia	Kazakhstan	Montenegro	Kenya
		Thailand	Turkey	Kyrgyzstan	Niue	Madagascar
		Timor-Leste	United Arab Emirates	Latvia	Panama	Maldives
		Viet Nam	Yemen	Macedonia	Papua New Guinea	Mozambique
				Palestine	Poland	Namibia
				Russia	Romania	Nigeria
				Serbia	Samoa	Rwanda
				Tajikistan	Slovakia	Senegal
				Turkmenistan	Slovenia	Seychelles
				Uzbekistan	Suriname	Sierra Leone
					Trinidad and Tobago	Somalia
					Ukraine	South Africa
						South Sudan
						Sudan
						Tanzania
						Togo
						Zambia
						Zimbabwe

Table 3. Summary of variables

Variable name	Description	Source
Degree of internationalization (DOI_{BRI})	A multi-item index that measures the level of international involvement of Chinese MNCs. It is a	WIND, CSMAR

	linear addition of 4 variables FSTS+OSTS+PDIO+GD	
FSTS	Percentage of foreign sales to overseas sales	WIND, CSMAR
OSTS	Percentage of overseas subsidiaries to Total subsidiaries	WIND, CSMAR
PDIO	The degree to which the operations of the firms are spread along psychic zones	WIND, CSMAR
GD	The variable captures extensity of international activities	Official OBOR website

Table 4. Descriptive Statistics POEs vs SOEs

Variable	Combined mean	SOEs (AVG)	POEs(AVG)
FSTS	0.2280	0.1691	0.2908
OSTS	0.2461	0.1753	0.3218
PDIO	0.1549	0.1893	0.1181
GD	0.0406	0.0605	0.0194
DOI_{BRI}	0.6698	0.5944	0.7503

Table 5. Overall Descriptive statistics

Variable	Observations	Mean	Std. Dev.	Min	Max
DOI_{BRI}	91	0.6698	0.4515	0.1119	2.1108
FSTS	90	0.2305	0.2603	0	0.9989
OSTS	91	0.2461	0.2911	0	1
PDIO	91	0.1549	0.1035	0.1	0.5
GD	91	0.0406	0.0697	0.0119	0.4047

Table 6. Correlation Matrix

	DOI	FSTS	OSTS	PDIO	GD
DOI	1				
FSTS	0.7701	1			
OSTS	0.7721	0.3685	1		
PDIO	0.1788	-0.1008	-0.2	1	
GD	0.1588	-0.1012	-0.21	0.855	1
