# Supplementary material for: The degree of internationalization of Chinese Multinationals along the belt and road initiative countries
Source: PLoS One. 2020 Jul 30;15(7):e0236636. doi: 10.1371/journal.pone.0236636 (PMC7392271; doi:10.1371/journal.pone.0236636)
Supplement: Attachment — Submitted filename: Response to Reviewers.docx [file pone.0236636.s004.docx]

We are grateful for the helpful feedback by the Academic editor and Reviewers that helped us to improve the quality of the manuscript. We carefully responded to all points and have modified the manuscript accordingly.

**1. Response to Academic Editor.**

*“The paper is interesting. However, there are important parts to make clear and complete and numerous writting mistakes. Also there are too numerous acronyms must be recalled regularly. Please answer carefully to the numerous remarks of the reviewers.”*

We appreciate the positive assessment of our work. The paper has been revised and reformatted. Furthermore, care has been taken, to as much as possible, do away with acronyms without affecting the paper. For instance acronyms have been significantly culled from the introduction, Discussion and Conclusion sections, as well as in the main text (e.g. P.21, line 447; P.28, line 571,575; P.30, line 614). In addition in the main text the acronym “MNCs” has been replaced with the word “multinationals”, only retaining it in tables and keywords. Similarly acronyms have been removed from the abstract.

**2. Response to Reviewer #1:**

*“This paper claims private firms have higher degree of internalization rather than state owned Chinese MNCs along the Belt and Road countries. The topic is novel and the conclusion is interesting. Within the particular definition of BRI countries in this paper, the methodology is sufficient to allow the reproduced experiment. However, several unsolved issues still exist in this paper:”*

***Literature*** *“Previous literature on the ownership of Chinese MNCs along BRI countries can be included, such as 10.13529/j.cnki.enterprise.economy.2018.11.002, which discusses the discrimination toward state owned capital of SOEs in BRI area.”*

Thank you for this suggestion and providing a fresh perspective.

***Data & Methodology*** *“Actually, the B&R initiative does not have a clear definition of its boundary yet. What are the specific area/countries included in the paper?”*

Thank you for pointing this out. The countries included are based on the official Belt and Road website (<http://yidaiyilu.com.gov.cn>) as at the time of writing the paper in December 2018). We have included a citation in the first paragraph of the introduction to clarify this (*Profiles -Belt and Road Portal*, 2018) (P.2; line 46), as well as in the methodology section (P.22, line 453)

*“The numbers and sizes of SOEs and POEs are not the same, so they may not be comparative without any distinction.”*

You are correct indeed, and we acknowledge that the number and sizes are not the same. However the variable of concern is ownership. Explicitly, whether the firm is owned by the state or not. This is a categorical variable and not subject to firm size. In addition, we do note in our discussion the distinction that state owned firms tend towards the huge infrastructural projects relative to their private counterparts. Furthermore the literature reports mixed findings on the relationship between size and degree of internationalization (Pangarkar, 2008).

*“Similarly, in table 3，only using the number of countries/firms to describe the PDIO and GD is not sufficient, at least the volume of countries/firms should be considered”*

We follow the literature in measuring geographic dispersion as a ratio of the potential number of countries in which a firm can have operations and the actual number of countries in which the firm has operations(Ietto-Gillies, 1998). This in this case corresponds to all countries in the belt and road initiative. Furthermore, Ietto-Gilles notes

“There might be a degree of arbitrariness in the choice of this number;

however, such a choice does not affect the results since the exact value of the

denominator in the index is not very relevant. What is needed is a datum which

expresses the potential number of countries and which can be kept constant for all companies, at any given period of time.” (Ietto-Gillies, 1998, p. 26)

Similarly in adopting the same approach for the measure of psychic dispersion, we conform to the literature as both Sullivan(1994) based on psychic zones as well as Fisch and Oesterle(2003) measured cultural and psychic dispersion in like manner.

We would also like to point out, since both PDIO and GD are dispersion measures, they are designed to capture the extensity of activities and not the intensity. In other words the breadth and scope of activities and not magnitude.

*“In table 3, what is the difference between foreign sales and overseas sales in the indicator FSTS?”*

Thank you for this observation. It was meant to read overseas subsidiaries. The error has now been fixed.(p.13)

***Writing*** *“Several spelling and grammar mistakes make some paragraphs ambiguous: 1) “However, dspite its economic and politiacal significance of the BRI, there are only a few empirical studies on the internationalization activities of Chinese MNCs in countries along the” in p9.*

*“…because the quantitative studies have mainly examined the causal relationship between the BRI and changes in the levels of Chinese OFDI, a preference for the Difference in differences thechnique for data analysis [4,5,8] was observed.”*

Thank you for this observation, we acknowledge the writing errors. All the errors have been corrected.

*“In table 3, DOI is simple multiplication of 4 items. The four items may not be equally effective in DOI, and then the different coefficients should be considered.”*

We follow Sullivan (1994, 1996) in using a composite index that is based on a linear addition of the different variables measuring various dimensions of internationalization.. Furthermore, equal weights are assigned to each item because the core thrust of our argument is that the different aspects of internationalization are given adequate consideration. Sullivan (1996) addresses the merits of using this approach rather than varying the weights assigned to each item.

**3. Response to Reviewer #2:**

*“The research presents an interesting and important subject in the complex area of internationalization strategies of BRI countries. It attempts to assess the degree of internationalization (DOI) of Chinese firms along the Belt and Road initiative countries and then to find the relation of firm ownership to DOI. But, current manuscript needs some corrections to present the research process and results clearly. Please see the below comments”*

***Abstract:***

*“it is better to explain the variables of analysis and then the complete results of the research rather than just indicating the first hypothesis.”*

Thank you for this suggestion. The abstract has been rewritten to include a mention of variables and dimensions of internationalization measured as suggested, and the key findings.

***Introduction****:*

*“For the reference 9, authors referred to the conditions of internationalization (citing Dunning), however as it is the first time in the manuscript it is necessary to explain these conditions. It is explained on the page 6. The same is for all abbreviations which are introduced for the first time in the manuscript. This will improve the readability of the context.”*

This has been rectified via a short description of the antecedents of internationalization (P.3, line 75-80)

*“on page 3, in contrast to the abstract, results should not be explained in the introduction part. Rather than giving unnecessary information, it is better to explain the case study (BRI project), the problem statement, the importance of doing research in this case study and on this subject, main questions and aims of the research, hypothesizes and the procedural framework of the research.”*

The background of the BRI was provided in the introduction along with the problem statement, significance of the study as well as the aim of the research in the last paragraph on page 3 as well as the first paragraphs of page 4 (p.3 lines 58,67; p.4 lines 80,92; P.5 line 98). Also, page 5 contains the literature review on belt and road initiative research.

*“The statement of “Lending credence to the view that under certain conditions, the behaviours of SOEs and POEs in terms of international activities converge and become indistinguishable [7,17]. Suggesting that perhaps along the BRI, the advantages bestowed by state ownership are irrelevant to successful internationalization” is a critical and interesting statement in this research and in better to be deeply explained in the proper part of the manuscript (in the part of results and not introduction)”.*

Thank you for this comment and observation. This excerpt has now been moved to the discussion section (p.28, line 566)

***Theoretical Framework*** *“For the first part: the previous studies on BRI considering OFDI trends, different economic activities and economic evolution related to implementation of BRI, are reviewed. However these explanations do not provide new insights for the theoretical framework of this research. They can be helpful to justify the novelty and necessity of this research toward the previous ones”*

You are indeed correct; the aim of the review was to show the gap in the current belt and road research. We identify the missing firm level perspective in extant BRI research, as Internationalization per se has not been investigated, consequently the potential of the BRI to extend internationalization research is highlighted,(p.7 line 142).

*“In the second part, authors attempted to review the theories of firm internationalization. However instead of first defining the concept and its different determinants, it discussed the internationalization of Chinese firms providing fragmented critiques from different approaches without making clear what exactly they are. (e.g. discussing OLI approach for CFI without explaining Dunning’s approach). This trend is continued in the methodological part and even in the conclusion. So it is strongly recommended to reorganize the theoretical part; first define the concept of “firm internationalization” based on different theories, and clarify its similarities and differences from different conceptual lenses”.*

We appreciate this suggestion. These recommendations have now been incorporated into the paper via paragraphs 1-3 on page 8.

*“Also rather than considering the Dunning’s theory (OLI), or Rugman’s (for FSA, CSA) and Haier and Galanz’s study occasionally, just start with the theories of macroeconomic and then go to the details of firm-base perspectives such as the Uppsala model, the eclectic paradigm, network theories and etc. and shape the theoretical implications of “firm-internationalization”. And now discuss the way that Chinese firms are internationalized. “*

We appreciate and acknowledge your observation and it has been included in the paper, (P.8 lines 164,175; p.9,lines 188,196; P.10 line 222).Thank you.

*“This manuscript contains lots of errors in its writing. some are colored in yellow in the attached file.”*

These have all been corrected.

***Methodological Framework***
 *“The last paragraph on page 12, defining the international firm, again have to be transferred to the theoretical part.”*

Thank you for this observation, it has now been implemented.

*“The last paragraph again discusses the difference of this study with the previous studies on BRI, just based on its firm-level scale of the analysis. It has the potential to clarify its contribution to the theories of firm internationalization as well. In the third part, authors tried to discuss the FI measurement. Again considering the Uppsala model and methods introduced by Sullivan and Ramawamy et al. however references 14 and 15 are unrelated to the previous methods of measurement. But the point is that this part should be integrated either with the conceptualization part or with the methodological part for building the indexes of analysis. The fourth part: hypothesizes, can be explained in the problem statement.”*

We appreciate your suggestions and they have been incorporated into the paper. The third part has been done away with and integrated into both the introduction and the methodology sections. Regarding the Hypotheses we are of the opinion that retaining it in the final section of literature review improves the flow and readability of the article. Consequently it was included there.

*“In the last paragraph of the “variables”, on page 15, the three dimensions of structure, attitude and performance are discussed but the GD and its relation to the main determinants of IF is neglected”.*

Thank you for this observation. The GD has now been added to the discussion.

*“Table 3 has to include the exact formula and method of calculation of each variable and the way to discount the scale differences of these non-weight combination indexes.”
“How the variables of GD and PDIO are build and how do they differ from each other.”*

Table 3 has now been updated with the calculation methods.(p.22)

***Results*** *“Before presenting the results, it is better to explain the descriptive features of the variables in two groups of firms. For example, in each group, private or public, for each indexes, which firms have the higher and lower values. What are their characteristics, their maturity, the number of subsidiaries, the share of foreign investments, the economic activities (service, production, infrastructure, etc.). And in which BRI countries these POEs and SOEs have the most subsidiaries, trades and why.”*

*“In this part the authors just discussed the result of first hypothesis and the second one is neglected.”*

Thank you for this suggestion. A presentation of the descriptive statistics has now been included. As well as two tables displaying the statistics for the individual variables for the state owned enterprises and private owned enterprises respectively (p.23; p.24). In addition, a table with all the firms and their respective scores has been included in the appendix.
